# Breast cancer recurrence after reconstruction: know thine enemy

**DOI:** 10.18632/oncotarget.25602

**Published:** 2018-06-12

**Authors:** Elizabeth A. Brett, Matthias M. Aitzetmüller, Matthias A. Sauter, Georg M. Huemer, Hans-Günther Machens, Dominik Duscher

**Affiliations:** ^1^ Department of Plastic and Hand Surgery, Technical University of Munich, Munich 81675, Germany; ^2^ Section of Plastic and Reconstructive Surgery, Kepler University Hospital Linz, Linz 4020, Austria

**Keywords:** breast cancer recurrence, reconstructive surgery, flaps, fat grafting, breast implants

## Abstract

Breast reconstruction proceeding cancer treatment carries risk, regardless of the type of surgery. From fat grafting, to flap placement, to implants, there is no guarantee that reconstruction will not stimulate breast cancer recurrence. Research in this field is clearly divided into two parts: scientific interventional studies and clinical retrospective evidence. The reconstructive procedure offers hypoxia, a wound microenvironment, bacterial load, adipose derived stem cells; agents shown experimentally to cause increased cancer cell activity. This is compelling scientific evidence which serves to bring uncertainty and fear to the reconstructive procedure. In the absence of clinical evidence, this laboratory literature landscape is now informing surgical choices. Curiously, clinical studies have not shown a clear link between breast cancer recurrence and reconstructive surgery. Where does that leave us? This review aims to analyze the science and the surgery, thereby understanding the oncological fear which accompanies breast cancer reconstruction.

## INTRODUCTION

Surgical caution surrounding breast cancer reconstruction is understandable and rational. Defining a site as ‘cancer free’ can be inaccurate, as recurrence is common. It is estimated that up to 40% of all breast cancer patients will experience relapse, the highest risk of relapse being within the first 1–3 years post reconstruction [1]. After treatment, many patients take measures to reduce recurrence risk into their own hands. This includes decreasing alcohol intake [2], prolonged nightly fasting (13 hours) [3], and even green tea consumption [4], which have all been linked to reducing recurrence. However, chance of recurrence is the constant enemy of post-cancer patients, no matter what lifestyle measures are taken. For this reason, reconstruction is frequently forgone. A review of 125 breast cancer patients revealed that while 89% are afraid of the appearance post-op, 63% are still afraid their reconstruction would mask recurrence [5]. As such, breast cancer reconstruction holds uncertainty for both patients and surgeons. Here we review the evidence behind the ‘fear’ of surgically creating a state/condition that will cause recurrence secondary to reconstruction.

## UNDERSTANDING THE ZONE TO BE RECONSTRUCTED

Up until the 1980 s, it was not considered safe to reconstruct a breast until 2 years after the original mastectomy [6]. Nowadays however, there is a wealth of evidence supporting immediate reconstruction and the oncologic safety thereof. Reconstruction may be immediate, delayed-immediate (using a tissue expander), or delayed [7]. As a departure from Halsted’s radical mastectomy of the late 1800 s [8], the trend of nipple sparing mastectomy (NSM) and skin sparing mastectomy (SSM) has brought reconstruction from general to plastic surgery. Some retrospective studies suggest that NSM does not correlate with cancer recurrence [9, 10], while some recommend extreme caution [11, 12]. As for SSM, ‘skin sparing’ varies from patient to patient, making it difficult to apply a standard to retrospective literature [13]. However, it was reported that 0 of 44 patients with ductal carcinoma *in situ* (DCIS) relapsed upon SSM, while 10 out of 177 with invasive ductal carcinoma presented relapse within 10 years [14]. When it does occur, DCIS relapse with SSM has been correlated with the young age of the mastectomy patients [15].

Looking microscopically, there are differences between the microenvironments of full and partial mastectomies. A full mastectomy will not have any ductal or glandular tissue remaining. Similarly, large parts of the skin including the nipple will be removed. The reconstructed area then assumes the microenvironment of the transplanted flap/graft/acellular dermal matrix (ADM) [16]. Meanwhile, the microenvironment of the partial mastectomy will feature surgically disrupted lobular tissue, creating the possibility of benign to malignant duct conversion [17]. Ligation of vessels can lead to downstream hypoxia, a known driver for tumor development [18]. Skin that is spared presents immunological refuge for remaining cancer cells, which preferentially seek shelter in proximal dermis [19]. A modified radical mastectomy has markers present in drain fluid which can indicate status of microenvironment (IL-6 and TNF mark a healthy healing process, and IL-4 and interferon-g indicate post operative necrosis and seroma) [20]. There is no such standard available for partial mastectomy, given the variability of underlying tissue processes.

## EVIDENCE FOR BREAST CANCER RELAPSE POST RECONSTRUCTION

**Figure 1:**
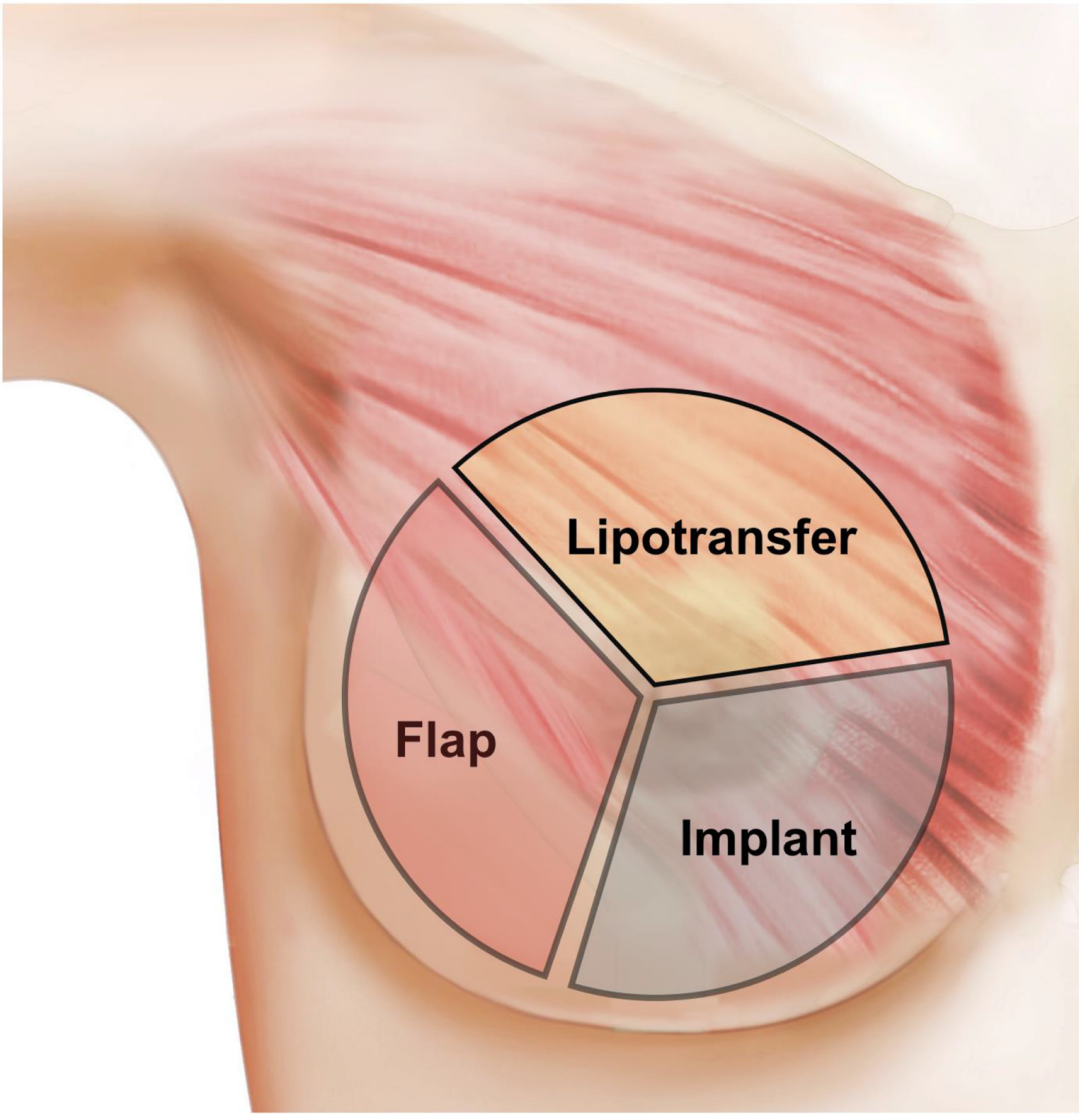
This schematic summarizes the main surgical mechanisms of reconstruction (flap, lipofilling/fat grafting, implant placement) These can be performed in isolation, but are often used together as combination surgery.

Cancer relapse is a multivariate phenomenon. ‘Minimal residual disease’ (MRD) is a term encompassing local, circulating and disseminated tumor cells [21]. Cancer type, stage of development, previous treatment history, age, and sensitivity of the diagnosis all play a role in portending the cancer relapse [22, 23]. Below, we briefly outline the relationships of reconstruction methods with relapse occurring after surgical reconstructions (Figure 1).

### Evidence for breast cancer relapse after flap reconstruction

There is long standing evidence for immediate reconstruction post mastectomy with flaps and the related absence of relapse. Specifically, myocutaneous flaps (rectus abdominis, latissimus dorsi (LD)) are quoted as safe bloc transplants in a post cancerous setting [24]. Immediate primary reconstruction with LD flaps in 51 patients were followed out for almost 45 months, with relapse showing in 4/51 [25]. Similar findings were reported in 2013, where immediate reconstruction via SSM and transverse rectus abdominus muscle (TRAM) flaps showed 55 of the 249 patients relapse, 33 with lung metastases [26]. Even in late stage patients, early reconstruction with flaps is lauded as an oncologically safe procedure [27]. Due to excessive donor site morbidity, the DIEP is preferable to the TRAM [28]. In a comparison with the older TRAM flap, the DIEP has not exhibited greater cancer recurrence [29]. Recurrence with DIEP flaps has been reported on the ipsilateral side rather than contralateral, 3–5 years post reconstruction [30]. Importantly, in a combination therapy of delayed DIEP and lipofilling, there was observed no significant increase in relapse between DIEP/fat grafting and delayed DIEP alone [31]. Myriad other free flaps exist as reconstructive options for breast cancer; involving the gracilis muscle (transverse upper gracilis, TUG), abdominal (superficial inferior epigastric artery, SIEA), and fascio-cutaneous infragluteal (FCI) [32], but are rather under-described in terms of cancer stimulation during reconstruction.

### Evidence for breast cancer relapse after implant reconstruction

The literary landscape of breast cancer recurrence stimulated by implants is sparse. Breast implants carry risk of cancer development in healthy patients, lending natural concern to the field of cancer reconstruction by implants [33]. Distal pathologies caused by the implant are generally instigated by migrating silicone which typically settles in pectoral girdle nodes, causing lymphadenopathy [34]. Certain existing clinical data indicates no causal relationship between implant use and cancer recurrence in breast cancer patients [35, 36]. A study involving 176 subjects and matched controls (mastectomy patients with, mastectomy patients without implant) showed a closer association between implant failure and scleroderma, rather than recurrence [37]. Anaplastic large cell lymphoma (ALCL) is a concern associated with breast implants. According to the World Health Organization, ALCL is not breast cancer, but a category of T-cell lymphomas, characterized by cellular CD15-/CD30+/CD40+ expression and anaplastic morphology [38, 39]. The cells make and survive in an immune privileged peri-implant fibrotic capsule [40]. ALCL presents initially as seroma formation between the implant and the fibrotic capsule. Early detection is followed by removal of implant and capsule, but if the seroma has breached the capsule, systemic chemotherapy becomes advisable [41]. So far, the literature seems unusually unified in presenting rougher implant surface as a cause for ALCL [42, 43]. This poses interesting questions regarding increased biofilm infection on the large surface area of textured implants versus smooth; identified as a precursor to ALCL [44] [45]. Speaking in micromorts, the unit measure for risk of death, dying of breast implant associated-ALCL is 0.4. To contextualize, driving for eight hours has a micromort value of 16; 40 times the risk of BIA-ALCL death [46].

### Evidence for breast cancer relapse after fat grafting

Lipofilling or ‘fat grafting’ is a commonly used technique to enhance cosmetic results of breast reconstruction and sometimes even as the main reconstructive procedure. It involves liposuction of peripheral fat, usually from the abdomen, followed by subsequent centrifugation/phase separation to isolate the layer of tissue to be grafted [47]. It is an especially attractive solution to asymmetry/volume loss given the simplicity of technique and associated lack of scar. However, fat grafting is an imperfect tool. Cysts and ‘suspicious nodules’ may be observed sonographically post-op, resulting in excision of grafted tissue [48]. The oncologic safety of fat grafting post cancer treatment was a point of discussion in 2009 by the American Society of Plastic Surgeons. In a publication called “ASPS Fat Graft Task Force” [49], the society investigated the existing literature surrounding recurrence and lipofilling. However, the work is criticized for lack of attention paid to the trophic cancer milieu; there is no direct discussion on the impact of transplanted adipose tissue on existing cancer cells [50]. For instance, there is evidence of a cancer feedback loop involving lipogenesis, whose direction is unclear. Cancer tissue exhibits aberrant lipid biosynthesis ancillary to cancer proliferation via fatty acid synthase (FASN) [51]. The other direction, altered lipid synthesis may induce tumor angiogenesis [52]. It appears the role of lipid metabolism in cancer development may be greater than initially assumed, per metabolomic genome studies of nascent and advanced breast cancer [53].

In the 8 years which have elapsed since the ASPS report, more detailed clinical evidence has been provided on the topic of recurrence post-lipofilling. A study published in 2016 analyzed 719 breast cancer patients whose treatment was either segmental or total mastectomy, and whose reconstruction involved lipotransfer. The 670 matched controls were patients who had identical surgical treatment, and a reconstruction which did not feature lipofilling (e.g. flap/implant). The study saw no associated increased risk of cancer recurrence with lipotransfer when compared to the controls [54]. Another retrospective study published 4 years earlier reached the same conclusion; lipotransfer for repairing tissue deficit post-lumpectomy or –mastectomy in 321 patients saw recurrence in 27; a statistically insignificant indicator of cancer recurrence [55]. Seeing the small and satellite nature of the aforementioned studies, a meta analysis was published in 2017 delineating CRAFT (Cancer Recurrence After Fat Transfer), a numerically viable framework established for a multi-center case cohort study on breast cancer reconstructions [56]. An important inclusion criterion for the subjects in this study was immediate reconstruction post mastectomy. The authors did not observe increased risk with fat transfer in these patients.

However, in 2013, a self-acknowledged preliminary study showed patients with specifically ductal or lobular intraepithelial neoplasia having an increased risk of local recurrence with lipofilling [57]. The authors hypothesize that the low number of acquired genetic lesions on the ductal or lobular neoplastic cells means they are more receptive to paracrine signaling from grafted fat, as opposed to invasive breast cancer cells whose DNA is highly mutated, and less reactive to extracellular signals. With that in mind, it is of particular interest that the chief impact of adipose derived stem cells in the fat graft is paracrine [58]. A new approach harnessing these ambivalent trophic effects is cell assisted lipotransfer (CAL). The process of CAL entails grafting fat which is enhanced with a surplus of the patient’s own adipose derived stem cells (ASCs) [59]. It is important to note the paucity of oncological studies in this field, given that the skepticism and lack of knowledge has significantly halted the clinical implementation of CAL. In a retrospective study of CAL fat grafting clinical papers, there is quoted a high level of bias, absence of control groups, and widely ranging variation among studies, making parallel comparisons impossible [60].

## WHICH FACTORS MAY DRIVE RECURRENCE?

**Figure 2:**
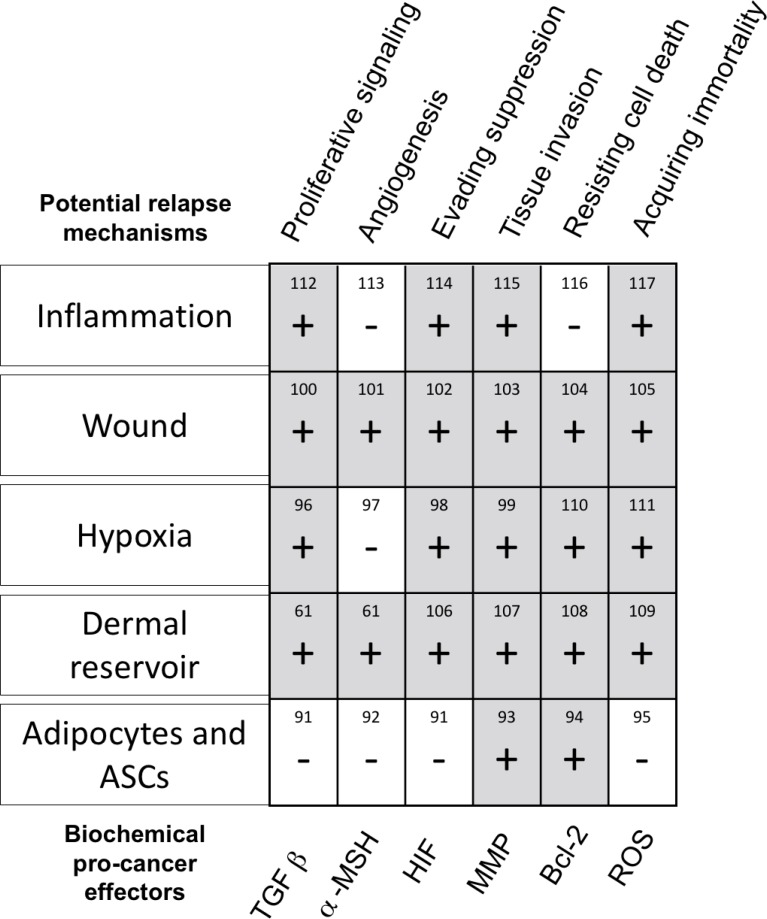
Breakdown 5 potential biochemical mechanisms which may cause relapse, and a chart of up/down -regulated pro-cancer factors (up = grey, ‘+’, down = white, ‘−’) Pro-cancer factors (diagonal, bottom of chart) were selected based on the 6 commonly accepted hallmarks of cancer (diagonal, top of chart) [90–117]. Taken in totality, it is noted that the wounds and dermal reservoirs possess the most potential relapse mechanisms, with most upregulated options for oncogenic signaling. Adipocytes and ASCs have the fewest upregulated pro-cancer genes. References for cancer and relapse literature are numbered within the table.

Clinical observations and scientific research of recurrence are not perfectly in sync. However, a range of scientific studies point at cancer circuitry within the reconstructive procedure (Figure 2). Below, we isolate the existing literature that shapes understanding of cancer relapse after reconstruction.

### Dermal reservoir of cancer cells

The skin has been quoted as an immune privilege area, in ways not dissimilar to the brain. Hair follicle Antigen Presenting Cells (APCs) lack MHCII. Potent immunosuppressive factors like TGFB1 and a-melanocyte stimulating hormone (a-MSH) are present in the dermis [61]. These factors create an environment which provides shelter for cancer cells during treatment [19]. A separate aspect inherent to preserving skin margins (as in SSM), is also the potential remaining breast tissue adherent to the hypodermis, allowing cancerous breast tissue to remain unintentionally [62].

### Systemic blood profile alterations

It is important to acknowledge the changing cytokine profile of the cancer patient undergoing reconstructive surgery, as circulating levels of anti-apoptotic or pro-angiogenic factors could serve as triggers for remaining cancer cells. A hematological study run on 82 breast cancer patients at time points one day before and 5 days after their TRAM flap reconstructive surgery revealed a dip in pro-angiogenic growth factors until day 5, when they began to climb again. The authors suggest choosing a time for reconstructive surgery when the blood chemistry is not so advantageous for cancer [63]. It is commonly acknowledged that depleting a tumor of vascularity will impede its growth [64], thus there is danger of a surgically stimulated increase in angiogenic factors supplied to a starved, opportunistic tumor [65, 66]. Surgical tissue trauma presents the angiogenic switch as an explanation for tumor stimulation, resulting in a local increase in pro-angiogenic factors and a key decrease in angiogenic inhibitors like TSP-1 [67]. A mouse model of ovarian cancer lends some clarity on the potential mechanism of surgical stress on cancer; a laparotomy performed on mice 4 days after cancer cell inoculation revealed larger tumors compared to the anesthesia only group (no surgery). Moreover, Propranolol dosage lead to a complete block of surgical impact on tumor growth, indicating a role for b-adrenergic signaling in tumor response to surgery [68].

### Tissue macro/micro trauma

The act of surgery or the physical breakdown of tissue in the breast could be a cause for cancer recurrence. A 2016 study focuses on expired time post surgery as a function of breast cancer relapse. The authors choose date of reconstruction, and date of mastectomy as time zero for 2 different plots. They analyze existing literature and observe a similar bimodal set of peaks in both graphs, showing recurrence at 2 years post surgery, then again at 5–6 years post surgery. Both surgical procedures of reconstruction and mastectomy therefore appear to induce similar biological impacts on subclinical cancer [69]. An article in 2007 questioned the cellular culprit for the relapse upon physical disturb. Polyak *et al.* observed that the threshold for DCIS becoming invasive ductal carcinoma is the physical breakdown of the basement membrane and myoepithelial barrier of the cancerous milk duct. The nature of this research implies an actual mechanical disruption which is to blame for transition of cancer from benign to malignant [17]. The authors suggest that many DCIS remain dormant, do not need resection and yet are still resected. The act of surgical resection may lead to malignancy when otherwise they may have remained subclinical.

### Genetic mutation

A recent study in 2017 uses a transgenic mouse model of tamoxifen induction and subsequent inhibition of breast cancer, which leaves cancerous cells behind as a faithful recapitulation of human MRD. Gene transcriptional studies showed an increased abnormal level of lipid metabolism in the mammary epithelial cells of regressed tumors; the residual cells of the cancer. This led to local DNA damage due to oxidative stress, triggering oncologic gene mutation in the hormonally-induced expanding mammary cell population. Thus, inherent irregular energy metabolism within the remaining cancer could be an extracellular trigger for relapse through stimulation of oncologic gene mutation [70]. Healing the surgical incision created by the breast reconstruction is a wound microenvironment in which cells face hypoxia and DNA damage. Specifically, wound environments are a source of pro-inflammatory mediators and chemokines locally, and paracrine secretions can cause somatic gene mutation [71]. Clinical observations mirror this cellular mechanism. A regression analysis on five-year cancer survival post-reconstruction for patients without a wound complication was 89.2%, compared to 64% for patients with complicated, intractable wounds [72].

### Hypoxia in the reconstructed breast

Hypoxia is a process simultaneous with breast reconstruction. Flaps which are supplied solely by their anastomoses have graft borders which are not immediately supplied by native, ingrowing vessels [73]. Implants and expanders exert pressure on the breast boundaries, which decreases microvasculature efficiency, leading to hypoxia [74]. Adipocyte viability based on oxygen diffusion, independent of local HIF pathway activation, reaches 0.2 cm into the grafted tissue from it’s periphery [75]. In this hypoxic environment, there are multiple aspects which cancer may take advantage of. It is commonly accepted that tumor hypoxia correlates positively with increased invasiveness and metastatic ability [76]. As a baseline, breast cancer biopsies have been shown to contain increased levels of HIF-1, and that the overexpression is connected with increased metastatic risk and mortality [77]. HIF1 expression is directly upstream of L1CAM and angiopoeitin-like 4, which are responsible for lung metastasis of breast adenocarcinoma cells [78]. Using microarrays on biopsy samples from 512 patients, a clear upregulation of HIF-2, as opposed to HIF-1 is present in breast cancer. This offers a specific molecule which the authors propose as a prophylactic target to decrease harmful effects of hypoxia [18]. In terms of extracellular impact, HIF1 hydroxylates and stiffens collagen matrices. Matrix mechanical hardening has been proposed as a histopathologic prognosticator of recurring cancer [79].

### Adipose derived stem cells in grafted fat

CAL has been shown *in vivo* to elicit regenerative effects via vascularization of the fat graft in a situation of radiation therapy [80]. Studies have shown that ASCs in fat have the ability to withstand the initial hypoxic environment of the graft, and remain viable to recruit new vasculature. This is critical in avoiding necrosis of tissue and involution of grafted fat, both of which would defy the purpose of fat grafting [81]. However, it is the same mechanism for which CAL earns skepticism in planning cancer reconstruction. A more recent study examined the paracrine impact of ASCs on co-grafted breast cancer cells *in vivo.* Tumors were artificially created in mice by co-injection of breast cancer cells with ASCs, the latter of which had been harvested from human fat and passaged *in vitro* (P3 to P8). Cancer cell to ASC ratios ranged from 1:1 to 1:3, with larger tumors resulting from the increasing ratio. Tumors excised and lysed for chemokine analysis showed tumors had high levels of CXCL1 and CXCL8. The mechanism was elucidated further using shRNA to knock out CXCL1/8 in the supplied ASCs; a co-injection which resulted in a smaller, less vascularized tumor [82]. This type of targeted research is a step in the right direction when considering the safety of CAL in cancer patients.

## IS BREAST RECONSTRUCTION POST-CANCER SAFE, OR NOT?

While it appears that there are multiple mechanisms in the reconstruction which create a pro-oncogenic environment, their consistent impact has yet to materialize *in vivo*. Indeed, recent research focuses on the risk-reducing mastectomy (‘oncoplastic surgery’) as the first line of defense against recurrence [83, 84]. In one study regarding flap surgery, among a list of varied complications (infection, abscess formation, skin necrosis, hematoma), recurrence is not listed as one of them [85]. While delayed wound healing and re-operation rates were higher with implant reconstruction, there is no significant increased risk of recurrence with implant placement compared to mastectomy alone [86]. Recently, it was found that lipofilling to augment the shape of an LD flap resulted in seromas and wound dehiscence. However, the fat grafting brought no recurrence to any patient in the study [87]. These reports are snapshots of a wider sample of work, partly described in the body of this review, which all generally report similar outcomes.

Therefore, it seems that the main risks of reconstruction are complications of healing of the reconstruction, rather than ipsilateral, local cancer recurrence. This review has included conflicting literature of breast cancer recurrence and reconstructive surgery. An important distinction to look at while assessing recurrence, is if the tumor is a true recurrent tumor, or a new primary tumor. Existing work has defined the ‘true recurrent’ as a tumor being within 3cm of the original tumor bed, and consisting of the same cell type as the original [88]. In this way, only true recurrent tumors are of relevance in reconstruction, as new primary tumors belong to phenomena not likely linked to the reconstructive procedure [89].

## CONCLUSIONS

Adverse events following reconstruction, such as seromas, hematomas, wound dehiscence, implant failure, and/or skin necrosis, can compromise the success of breast reconstruction. Patients are not guaranteed ‘safe’ from these complications. However, the complication of cancer recurrence seems to arise independent of reconstructive procedures. Scientific studies which disparage breast cancer reconstruction share the fact that they are not investigating why recurrence is happening, rather proposing mechanisms by which relapse may develop. As such, the field has become clouded by hypotheses and doubt. Since much of the surgical caution comes from studies based on oncogenic potential, rather than evidence, it must be concluded that breast cancer reconstruction is not directly linked to recurrence; that reconstruction is safe.

Current recurrence research, independent of reconstructive surgery, is focused on remaining cancer cell activity. By comparison, little attention is paid to the remaining stroma created by the cancer, and its power in corrupting local cells to stimulate recurrence. A valuable new line of research in this field should incorporate the remaining extracellular matrix, and the cellular response it elicits. Similarly, a rather unreported source of information is in the biopsy tissue of the recurrent tumor itself. An alternate approach to this field is reflecting on biopsy evidence, which can help guide scientific studies. As it stands, this field is fractured by conflicting research. When the existing clinical data is met with appropriate scientific questions, the uncertainty surrounding breast cancer reconstruction may be removed.
